# Delivery of modified mRNA encoding vesicular stomatitis virus matrix protein for colon cancer gene therapy

**DOI:** 10.1039/c7ra13656k

**Published:** 2018-03-28

**Authors:** Ke Men, Rui Zhang, Xueyan Zhang, Rong Huang, Guonian Zhu, Rongsheng Tong, Li Yang, Yuquan Wei, Xingmei Duan

**Affiliations:** State Key Laboratory of Biotherapy and Cancer Center/Collaborative Innovation Center for Biotherapy, West China Hospital, Sichuan University Chengdu 610041 People's Republic of China mendingbob@hotmail.com duanxingmei2003@163.com; Individualized Medication Key Laboratory of Sichuan Province, Department of Pharmacy, Sichuan Provincial People's Hospital Chengdu 610072 People's Republic of China

## Abstract

Plasmid DNA based gene delivery has been widely utilized among both pre-clinical and clinical gene therapy studies. However, therapeutic efficiency is usually limited by the size and potential immune-stimulation issue of plasmid backbone. As an alternative form of genetic material, chemically modified messenger RNA (mRNA) provides a promising alternative to plasmid DNA. In this work, an *in vitro* transcription mRNA encoding vesicular stomatitis virus matrix protein (VSVMP) was delivered by a cationic liposome–protamine complex, resulting in high mRNA transporting and expression efficiency. The liposome–protamine complex delivered VSVMP mRNA strongly inhibits the growth of C26 tumor cells through inducing apoptosis, while obvious tumor regressions were achieved on both abdominal cavity metastatic and subcutaneous xenograft models *in vivo* with high safety. Our results also demonstrated that the liposome–protamine–mRNA complex was as potent as its plasmid DNA counterpart, showing strong potential in further colon cancer therapy.

## Abstract 2

Liposome–protamine complex delivered VSVMP mRNA efficiently inhibits C26 colon carcinoma with safety, providing an alternative strategy for non-viral gene therapy.

## Graphical abstract

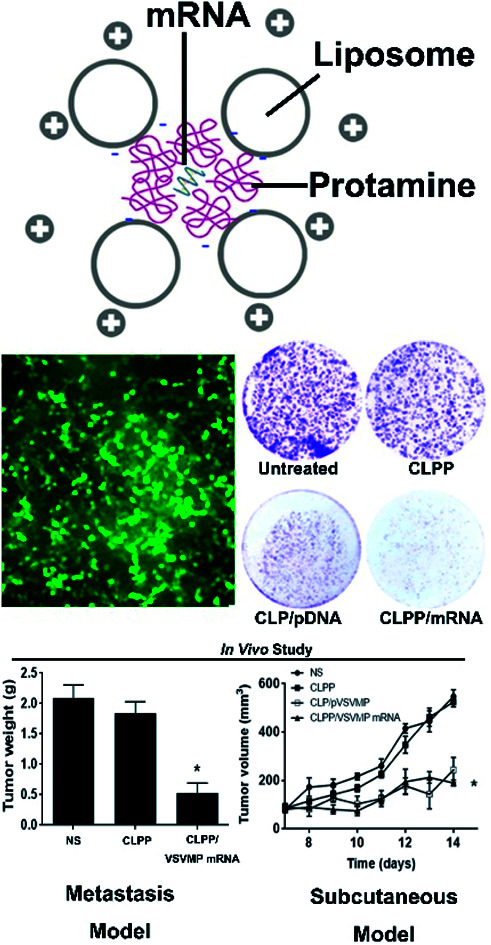

## Introduction

Cancer is one of the leading causes of death in both economically developed and developing countries.^1^ Colon carcinoma holds the second most common cause of death among cancers.^1,2^ Gene therapy with viral or non-viral delivery systems has been considered to be a useful strategy for cancer treatment. Although much progress on clinical application has been made within viral vectors such as adeno-associated virus (AAVs), and retrovirus, plasmid-based non-viral complex systems are always in great demand in pre-clinical research.^3–6^ Apoptosis is a physiological cell suicide program that is critical for the maintenance of healthy tissues.^7^ Inducing apoptosis in tumor cells by delivering suicide gene encoded plasmids has been proved to be efficient for cancer therapy. In our previous works, delivering apoptosis-inducing genes such as VSVMP and survivin-T34A has been evaluated on several cancer models and desired therapeutic effects were achieved.^8–10^ However, the delivery and expression efficiency of the therapeutic gene was highly restricted by the size of plasmids in certain circumstances. This always results in tremendous efforts and costs on optimizing delivery vectors. Meanwhile, the cytotoxicity and immune-stimulation issue of empty plasmid backbones could always be observed during experiment, which in turn interferes the evaluation of curing effect. Thus, developing alternative forms of therapeutic gene is crucial to further facilitate non-viral vector-based therapy.

As a natural product of genes, messenger RNA (mRNA) is a transient entity that mediates the translation of genetic information from DNA to proteins in cells.^11^*In vitro* transcribed messenger RNA (IVT mRNA) has been applied as an alternative therapeutic molecule to plasmid DNA in the field of cancer immunotherapy and stem cell-based biomedical research.^5,12–14^ Comparing to other forms of therapeutic genes, mRNA-based therapeutics have several advantages. Unlike plasmid DNA and viral vectors, mRNA do not integrate into the host genome, avoiding aberrant transcription and insertional mutagenesis.^15^ Its expression kinetics is predictable and consistent,^14,16,17^ while nuclear localization of mRNA is not required before rapid protein expression even in nondividing and hard-to-transfect cells.^18,19^ Moreover, mRNA is only transiently active and is completely biodegradable *via* metabolic pathways.^18^ What's more important, for specific gene expression, little elements are required for mRNA than plasmid DNA vectors, thus greatly lower the delivery difficulty and risks of side effects. These properties make mRNA a safe and attractive genetic material for gene-based therapy.^18^ However, what prevents mRNA from becoming a widespread therapeutic tool for gene therapy is its perceived instability, susceptibility to degradation, insufficient translatability and immune-stimulatory effects.^18,20,21^ As a result, substantial modifications have been invested to optimizing the structural of IVT mRNA including 5′ cap, 5′- and 3′-UTRs, the coding region, and the poly(A) tail.^11^ These efforts have overcome the aforementioned shortcomings with improved intracellular stability and translational efficiency.^22,23^ Nowadays, IVT mRNA has undergone extensive clinical or pre-clinical investigation in the fields of therapeutic cancer vaccination,^24–27^ cell programming^28–30^ and so on, demonstrating great potential.^18^

In this work, we attempt to evaluate the therapeutic effect of VSVMP gene in a mRNA form, and compare that with its conventional used plasmid counterpart. A cationic liposome–protamine complex will be utilized to deliver the *in vitro* transcription mRNA. Protamine has been reported to condenses nucleic acid, such as naked mRNA, into nano-sized complexes and protect it from nuclease degradation inside the lysosomes/endosomes, resulting in high expression efficiency.^31^ Meanwhile, cationic liposomes act as a conventional vector for efficient delivery the above complex. This strategy has been successfully applied for mRNA delivery in several reports including biomedical research and clinical trials.^32–35^ However, the expression efficiency is likely to be influenced by the length of mRNA, and delivering VSVMP gene in the form of mRNA has not been performed according to our acknowledgement. Furthermore, by mRNA administration, whether the anti-cancer ability or safety of VSVMP gene will be retained is still unknown. Thus, in this work, we attempt to delivery VSVMP mRNA by liposome–protamine complex for cancer therapy. The mRNA delivery efficiency will be evaluated through different aspects. We assume that liposome–protamine complex delivered VSVMP mRNA could efficiently inhibit C26 murine colon cancer with estimated mechanism.

## Methods

### Materials

DOTAP were purchased from Avanti Polar Lipids (Alabaster, AL). Cholesterol, 3-(4,5-dimethylthiazol-2-yl)-2,5-diphenyl tetrazolium bromide (MTT) protamine sulfate was purchased Sigma-Aldrich (St Louis, MO). All the other chemicals were purchased from Sigma-Aldrich unless otherwise mentioned. mMESSAGE mMACHINE™ T7 Transcription Kit and an MEGAclear™ Transcription Clean-Up Kit, OptiMem®, Lipofectamine® 3000, Dulbecco's modified Eagle's medium (DMEM) and serums were purchased from Thermo Fisher Scientific. CT26 *Mus musculus* colon carcinoma cell line (ATCC® CRL-2638™) and 293t human embryonic kidney cell line (ATCC® CRL-3216™) were purchased from the American Type Culture Collection (ATCC). The plasmid pVAX1-VSVMP expressing vesicular stomatitis virus matrix protein has been described previously.^10^ All plasmids were propagated in *E. coli* and purified by an EndoFree Plasmid Giga kit (Qiagen, Chatsworth, CA). BALB/c mice were obtained from Beijing HFK Bio-technology Co. Ltd. (Beijing, China) and maintained under specific pathogen-free conditions. All animal procedures were approved and controlled by the Institutional Animal Care and Treatment Committee of Sichuan University and carried out according to the Animal Care and Use Guidelines of Sichuan University.

### *In vitro* transcription of mRNA

VSVMP encoding mRNA was prepared by T7 polymerase-based *in vitro* transcription method. Briefly, the open-reading frame of the gene of VSVMP was amplified from pVAX1-VSVMP plasmid by PCR reaction with forward primer TAA TAC GAC TCA CTA TAG GGA TGA GTT CCT TAA AGA AGA TTC and reverse primer TCA TTT GAA GTG GCT GAT AGA ATC. The amplicons were used as templates for *in vitro* transcription using mMESSAGE mMACHINE™ T7 Transcription Kit. The mRNA transcription process was conducted according to manufacturer's manual. The prepared mRNA was further purified by using the MEGAclear™ Transcription Clean-Up Kit according to manufacturer's manual. The final products were quantified by spectrophotometry and analyzed by agarose gel electrophoresis to confirm the synthesis of full-length mRNA.

### Liposome preparation

Cationic liposomes (CLP) were prepared according our previous reports.^36^ Briefly, DOTAP and cholesterol (1 : 1, mol/mol) were co-dissolved in chloroform and solvent was removed under rotary evaporation. The lipid film is re-hydrated with distilled water under 50 °C to form cationic liposome solution with a final concentration of 10 mg mL^−1^. The size and surface charge of prepared liposomes were determined by Malvern ZS90 (Malvern, Worcestershire, UK) and stored in 4 °C for further use. For the delivery of IVT mRNA, cationic liposome–protamine complex (CLPP) was mixed with mRNA solution. Briefly, mRNA was first mixed with protamine sulfate solution (1 : 2 molar ratio). Then, cationic liposomes were added to the mixture in a ratio of 1 : 2 : 1 (liposome : protamine : mRNA, w/w/w) in distilled water, following by incubation at room temperature for 15 minutes. The cationic liposome/plasmid complex was prepared in similar method. Particularly, cationic liposome and plasmid DNA were mixed in a ratio of 5 : 1 (w/w).

### mRNA retarding assay

The mRNA binding ability of protamine–liposome complex to mRNA was evaluated by agarose retarding assay. The VSVMP mRNA delivered cationic liposome–protamine complex (CLPP/VSVMP mRNA) were electrophoresed on 1% (w/v) agarose gel for 30 min at 100 V. 1 mg of VSVMP mRNA was mixed with different ratios of CLPP. Gel was then stained with ethidium bromide (0.5 mg mL^−1^) and illuminated by a UV illuminator (Bio-Rad ChemiDox XRS, USA).

### *In vitro* transfection

24 hours before transfection, 293t or C26 cells were seeded into a 24-well plate at a density of 1 × 10^4^ cells per well in 0.5 mL of complete medium (DMEM containing 10% FBS). Enhanced GFP (EGFP) encoding mRNA (TriLink Biotechnologies, San Diego, CA) was used as a reporter gene. Particle equivalent to 1 μg of mRNA encoding EGFP was added to each well in the presence of OptiMEM medium. Polyethyleneimine (PEI25K), equal amount of liposome or protamine was used as a transfection control. The mass ratio of mRNA to PEI25K was and 1 : 1. The medium was then replaced with full medium 4 hours post-transfection. 12 hours or 24 hours later, pictures of each well were taken under microscope and the transfection efficiency was determined by flow cytometry (NovoCyte Flow Cytometer, ACEA Biosciences, USA).

### Real-time PCR

To determine the intracellular level of VSVMP mRNA, total RNA was extracted from C26 cells or tumor samples using TRIzol™ Reagent (Thermo Fisher Scientific, USA) and individual cDNAs were synthesized with a SuperScript II reverse transcriptase assay (Sigma-Aldrich). Real-time quantitative PCR was performed with a SYBR GreenER quantitative PCR SuperMix Universal kit (Sigma-Aldrich). Reactions were run with a standard cycling program: 50 °C for 2 minutes, 95 °C for 10 minutes, 40 cycles of 95 °C for 15 seconds, and 60 °C for 1 minutes, on an AB7500 real-time PCR system (Applied Biosystems, Foster City, CA). The PCR primers to detect VSVMP (forward: CGA GCG CTC CAA TTG ACA AA, reverse: TTT CCC TGC CAT TCC GAT GT) and GAPDH (forward; 5′-ATG GGG AAG GTG AAG GTC G-3′, reverse; 5′-TAA AAG CAG CCC TGG TGA CC-3′) were synthesized and purified by TSINGKE Biological Technology (Chengdu, P. R. China).

### Anti-proliferation assay

C26 cells were seeded into a 96-well plate with a density of 1 × 10^4^ cells per well. After transfection with mRNA encoding VSVMP, cells were subjected to MTT cell proliferation assay 72 hours post-transfection. After incubation, 20 μL of MTT solution was added to each well and incubated at 37 °C for 4 hours. The formazan was solubilized by adding 200 μL DMSO and shaken at room temperature for 30 minutes. The absorbance was read at 570 nm by the Spectramax M5 Microtiter Plate Luminometer (Molecular Devices, USA). Absorbance of untreated cells was considered as 100%.

### Clonogenic assay

Liposome complexes equivalent to 0.5 μg of mRNA was administered to 1 × 10^3^ C26 cells seeded in 6-well plate. 4 hours post-transfection, medium was refreshed with complete DMEM culture medium. The cells were continuing cultured for 2 weeks to form colonies. Colonies were washed with PBS for two times before stained with 10% crystal violet blue for 15 minutes. This assay was repeated for three times and the number of clones as well as inhibition rate in each well were then calculated.

### *In vitro* apoptosis assay

The cell apoptosis inducing ability of VSVMP mRNA delivering cationic liposome–protamine complex was investigation by flow cytometry. C26 cells were pre-seeded into a 6-well plate with a density of 5 × 10^4^ cells per well. After transfection with CLPP delivered VSVMP mRNA complex (1 μg mRNA per well), liposome delivered VSVMP plasmid complex (1 μg DNA per well), normal saline (NS) and null vectors (in equivalent amount with related complex) separately for 4 hours, the medium was replaced by full medium. 72 hours later, cells were stained with propidium iodide and Annexin V-FITC (Sigma-Aldrich). The apoptotic cancer cells were measured by flow cytometry (NovoCyte Flow Cytometer, ACEA Biosciences, USA).

### *In vivo* tumor inhibition assay

For abdominal cavity metastatic model, BALB/c mice of 6–8 weeks old were intraperitoneally injected with 1 × 10^5^ C26 cells. On day 3, mice were randomized into 4 groups (5 mice per group) and numbered. CLPP/VSVMP mRNA complexes equivalent to 10 μg of mRNA was prepared as aforementioned were injected intraperitoneally every day for 7 treatments. Mice receiving equivalent normal saline or liposome–protamine complex (CLPP) were regarded as control group. On day 20, all mice were sacrificed by cervical vertebra dislocation, and their tumors were immediately harvested, weighed, and analyzed. The volumes of ascites in each group were also measured and collected.

For subcutaneous tumor model, BALB/c mice of 6–8 weeks old were inoculated with 5 × 10^6^ C26 cells on right flank. When the average tumor volume reached 100 mm^3^, mice were divided into 4 groups randomly. CLPP/VSVMP mRNA complexes equivalent to 10 μg of mRNA or CLP/VSVMP plasmid complexes equivalent to 10 μg of DNA were injected intratumorally every day for 7 treatments since the tumor volume reached 50 mm^3^. Mice receiving equivalent amount of normal saline, liposome or liposome–protamine complex were regarded as control group. Tumor size was measured and animal weight was monitored every 2 days until all animals were sacrificed. Tumor volume was calculated as (1/2 × length × width^2^).

### Histological analysis

Tumor tissue harvested from *in vivo* inhibition studies and were fixed and embedded in paraffin. Wax-embedded tissue sections were dewaxed and rehydrated before staining with Mayer's HE. To analyze apoptotic cells within tumor tissues, sections were stained with DeadEnd™ Fluorometric TUNEL System kit (Promega) according to the manufacturer's manual. The fluorescent image from each group was acquired through a fluorescence microscope (Olympus, Japan). For CD31 staining, tumor sections were blocked and subsequently incubated with rabbit anti-mouse CD31 antibodies (Abcam, USA) at 4 °C overnight. Appropriate horseradish peroxidase-conjugated secondary antibody was then applied. The micro-vessel density was visualized and determined through a fluorescence microscope (Olympus, Japan).

### Statistical analysis

Data were expressed as the means with 95% confidence intervals. Statistical analysis was performed with two tailed *t*-test or one-way analysis of variance (ANOVA) using Prism 5.0c Software (GraphPad Software, La Jolla, CA). For all results, statistical significance was defined by a value of *P* < 0.05.

## Results

### Preparation and characterization of CLPP/mRNA complex

VSVMP mRNA was synthesized through a T7 polymerase-based *in vitro* transcription method based on previously constructed VSVMP encoding plasmid pVAX1-VSVMP. The capped RNA with poly(A) tailing was prepared according to manufacturer's manual. The yielding of IVT mRNA was 30 μg per reaction detected by spectrophotometric analysis at 260 and 280 nm. As shown in Fig. 2a, the IVT products were electrophoresed and visualized on agarose gels with a proximate length of 690 bases, which is consistent with the coding template of VSVMP gene.

**Fig. 1:**
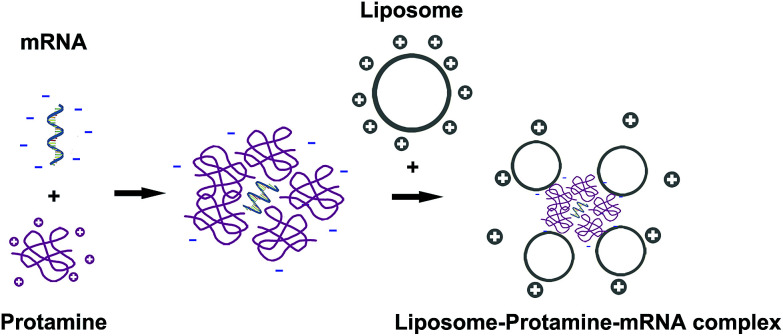
The preparation process of liposome–protamine–mRNA complex. mRNA was first condensed by protamine and then delivered by cationic liposome.

**Fig. 2:**
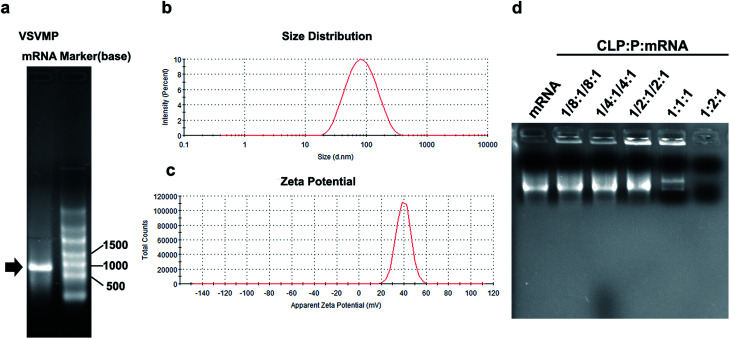
Characterization of CLPP/mRNA complex. (a) *In vitro* transcription of VSVMP mRNA; (b) size distribution of cationic liposome; (c) zeta potential of cationic liposome; (d) gel retarding assay of CLPP/mRNA complex.

In order to deliver IVT VSVMP mRNA, a cationic liposome–protamine complex system (CLPP) was constructed. The cationic liposomes were prepared using a thin-film method as previously described. As shown in Fig. 2b, the dynamic diameter of cationic liposome was 95.4 ± 3.5 nm with a polydispersity index of 0.22. The measured zeta potential was 39.7 ± 1.2 mV (Fig. 2c).

It has been reported that protamine could condenses nucleic acid into nano-sized complexes and protect it from nuclease degradation and thus facilitate gene delivery. For this reason, in our study, we first mixed mRNA with protamine sulfate solution (1 : 2 molar ratio) to well condense the nucleic acid. Then, cationic liposomes were added to the mixture in a molar ratio of mRNA : protamine : liposome = 1 : 2 : 1 followed by incubation (Fig. 1). In order to evaluate the binding ability of liposome–protamine complex (CLPP) to VSVMP mRNA, a gel retarding assay was performed. As shown in Fig. 2d, after electrophoresis, when the molar ratio of liposome : protamine : VSVMP mRNA was 1 : 2 : 1, no bright mRNA band was observed, suggesting that the negatively charged VSVMP mRNA was completely bond by cationic liposome–protamine complex through electronic interaction. This prescription ratio was chosen for further application in our study.

### *In vitro* transfection of CLPP/mRNA complex

**Fig. 3:**
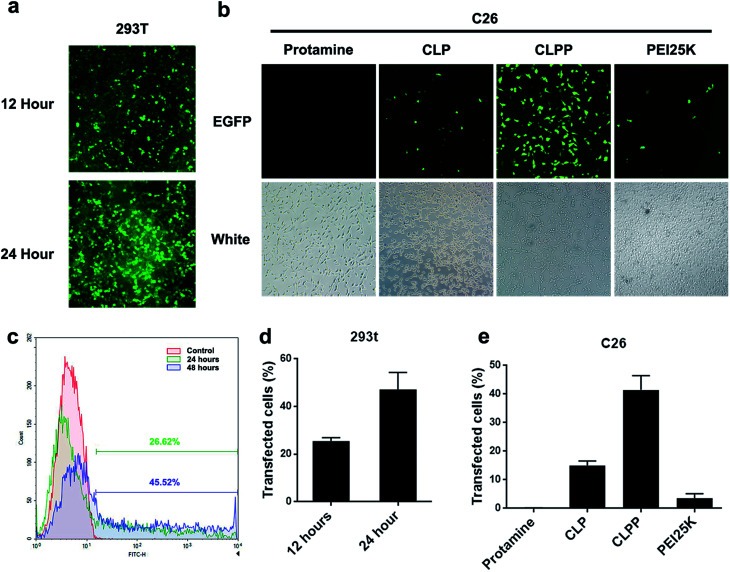
Liposome–protamine complex could efficiently deliver mRNA *in vitro*. The transfection efficiency of CLPP delivered EGFP mRNA on 293t cells in 12 and 24 hours analyzed by (a) fluorescent picture; (c) and (d) flow cytometry. The transfection efficiency of CLPP delivered EGFP mRNA on C26 cells analyzed by (b) fluorescent picture; (e) flow cytometry.

To further evaluate the mRNA delivery ability of liposome–protamine complex, their transfection efficiency was investigated *in vitro* on both 293t and C26 cells. As shown in Fig. 3a and b, both 293t and C26 cells could be efficiently transfected by CLPP/EGFP mRNA complex. This complex was able to transfect up to 25.54 ± 1.37% of 293t cells with a high expression level of EGFP after 12 hours. 24 hours post transfection, the ratio of cells expression EGFP increased to 47.17 ± 7.14% (Fig. 3a, c and 3d). Meanwhile, as to C26 cells, little fluorescent could be observed in either PEI (3.6% in average) or cationic liposome (15% in average) transfected well, while there was barely no fluorescent in protamine transfected well (Fig. 3b and e). These results indicated that CLPP complex was efficient in delivering and inducing EGFP mRNA expression in a short time. Although being potent in plasmid delivery, PEI25K and cationic liposome showed little mRNA transfection ability on C26 cells while protamine was almost incapable. Since the length of mRNA might directly affect delivery efficiency and that of EGFP mRNA used in our experiment was 996 bases long, our results further indicated that VSVMP mRNA (690 bases in total) delivered by CLPP would be highly expressed in cells within 24 hours. Meanwhile, it could be also observed from Fig. 3b that, comparing to PEI treated cells, little cytotoxicity was shown in CLPP group, suggesting high safety *in vitro*. Our results demonstrated that liposome–protamine complex could efficiently deliver VSVMP mRNA into C26 cells with safety.

### Anti-cancer ability of CLPP/VSVMP mRNA complex *in vitro*

**Fig. 4:**
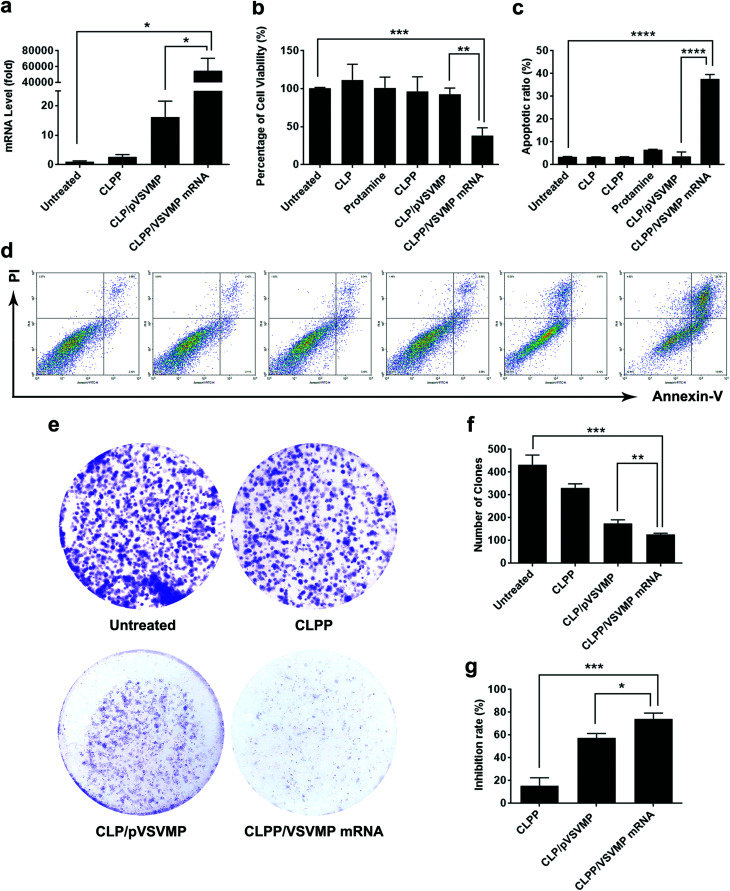
CLPP/VSVMP mRNA complex efficiently inhibit the growth of C26 cancer cells *in vitro*. (a) VSVMP mRNA levels in C26 cells after transfection; (b) inhibition effect of CLPP/VSVMP mRNA complex detected by MTT assay; (c) and (d) CLPP delivered VSVMP mRNA efficiently induced apoptosis in C26 cells; (e) inhibition effect of CLPP/VSVMP mRNA complex detected by clonogenic assay, the numbers of clones in each well were counted (f) and translated into inhibition rate (g).

The anti-cancer ability of CLPP delivered VSVMP mRNA was studied *in vitro*. We first evaluated the intracellular mRNA level of VSVMP gene after transfection. As shown in Fig. 4a, comparing to untreated group, 72 hours post transfection, a tremendous VSVMP mRNA level up to nearly 55 000 folds (*P* < 0.05) were detected in CLPP/VSVMP mRNA complex group, showing high mRNA delivery efficiency. Meanwhile, that of CLP delivered VSVMP plasmid group was much lower, with only 16 folds comparing to untreated groups.

To test its anti-proliferation effect on C26 colon cancer cells, a MTT assay was conducted. As shown in Fig. 4b, after 72 hours, obvious proliferation inhibition was observed in CLPP/VSVMP mRNA complex treated group, with an inhibition rate of 61.6% comparing to control group (*P* < 0.001). It indicated that CLPP/VSVMP mRNA complex equivalent to 0.5 μg of VSVMP mRNA was able to kill more than 50% of C26 cell *in vitro*. On the other hand, this effect was not reached by VSVMP plasmid group with an inhibition rate less than 20%, which showed significant difference comparing to mRNA group (*P* < 0.01). Meanwhile, VSVMP mRNA group without protamine was also inefficient in inhibition cell proliferation, which again indicated that protamine was crucial for the delivery of mRNA. What's more, our results also showed that liposome or protamine alone had little effect on cell proliferation, suggesting their potential safety.

The anti-proliferation capacity of CLPP/VSVMP mRNA complex was also evaluated by clonogenic assay. As shown in Fig. 4e, 14 days after transfection, much fewer clones could be observed in CLPP/VSVMP mRNA complex treated well than other wells. The number of clones in CLPP/VSVMP mRNA complex well was 127 ± 5 while that of NS control, liposome–protamine and CLP/pVSVMP was 433 ± 42, 331 ± 18 and 175 ± 16 (Fig. 4f), respectively. In this experiment, single C26 cells were cultured and grown into small clones which could be stained by crystal violet blue. The fewer clones being visualized implies stronger anti-proliferation capacity. Our results suggested that CLPP/VSVMP mRNA complex was more capable than equal amount of plasmid complex in treating C26 colon cancer *in vitro*, with an inhibition rate of 70.6% *versus* 59.6% (*P* < 0.05, Fig. 4g).

The apoptosis inducing property of VSVMP gene has been previously reported and applied in cancer therapy researches.^37,38^ To verify whether the anti-proliferation effect of CLPP/VSVMP mRNA complex on C26 cells was conducted by apoptosis inducing, cells in different treatment group was analyzed by flow cytometry with PI/Annexin V staining. According to our results, CLPP/VSVMP mRNA complex induced strong apoptosis in C26 cells (Fig. 4d). After been exposed to mRNA complex (1 μg mRNA) for 72 h, a total of 37.7 ± 1.8% of C26 cells were detected in early and late apoptosis phase (*P* < 0.001), while other groups including VSVMP plasmid complex failed to exhibit equivalent capacity (shown in Fig. 4c). Our results suggested that liposome–protamine complex could efficiently deliver VSVMP mRNA into C26 cells *in vitro*, inhibiting cell proliferation through apoptosis induction. Our results also suggested that CLPP/VSVMP mRNA complex was more potent in inducing apoptosis than equal amount of plasmid counterparts.

### CLPP/VSVMP mRNA complex inhibits C26 tumor growth *in vivo*

**Fig. 5:**
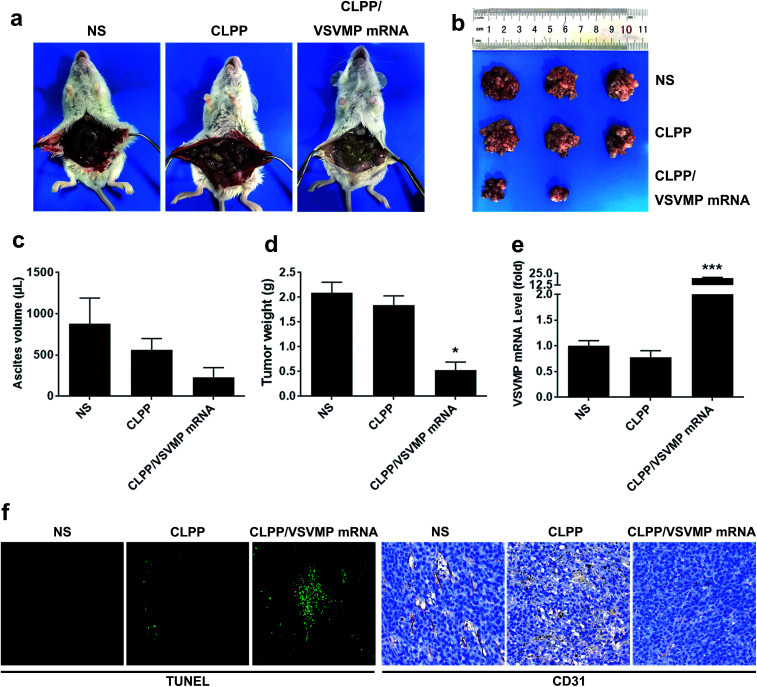
CLPP/VSVMP mRNA complex inhibit abdominal cavity metastatic tumor growth *in vivo*. (a) representative images of abdominal cavity metastases of C26 colon carcinoma; (b) tumor nodules harvested from each group; (c) average ascetics volume; (d) average tumor weight; (e) VSVMP mRNA level in tumor tissues; (f) apoptosis and vessels in tumor tissues detected by TUNEL assay (left) and CD31 staining (right).

The anti-cancer activity of CLPP/VSVMP mRNA complex was first evaluated on C26 abdominal cavity metastases model by intraperitoneal administration. Fig. 5a shows representative images of abdominal cavity metastases of C26 colon carcinoma in each treatment group. It was obvious that the mice treated with CLPP/VSVMP mRNA complex suffered mildest abdominal cavity metastases than other groups. As shown in Fig. 5b and d, compared with other group, VSVMP mRNA treatment group was much lower in metastases tumor weight (*P* < 0.05), with an average weight of 0.5 ± 0.2 g than those of NS group (2.1 ± 0.4 g) and CLPP group (1.8 ± 0.3 g). Meanwhile, as shown in Fig. 5c, there was also an obviously decrease in the ascites volume of CLPP/VSVMP mRNA complex treated mice. The volume of ascites in mice treated with mRNA complex was 0.2 ± 0.1 mL compared with 0.9 ± 0.4 mL in control group and 0.6 ± 0.2 mL in the mice treated with liposome–protamine. It can also be observed that the mice without mRNA complex treatment suffered from large volumes of blood-like ascites, suggesting serious tumor infiltrating and inflammation. These results indicated that CLPP/VSVMP mRNA complex efficiently suppressed tumor growth of abdominal cavity metastases *in vivo*.

**Fig. 6:**
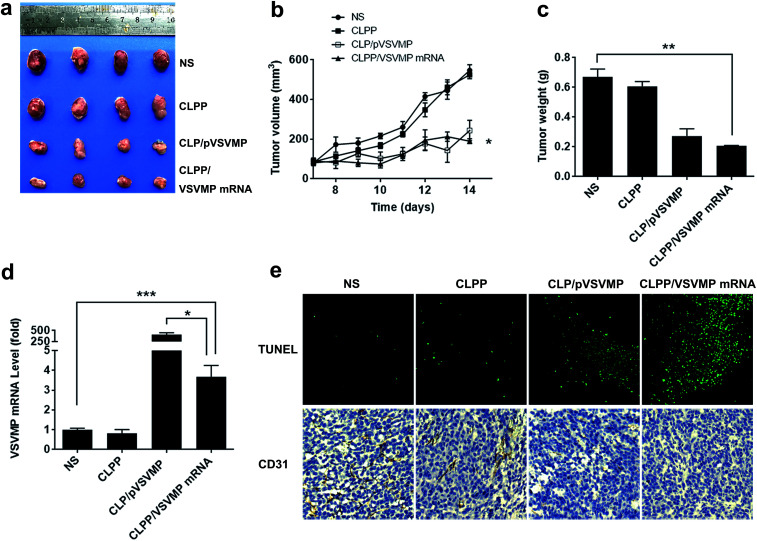
CLPP/VSVMP mRNA complex inhibit subcutaneous xenograft tumor growth *in vivo*. (a) Representative images of tumors of C26 colon carcinoma; (b) tumor growth curves of each group; (c) average tumor weight; (d) VSVMP mRNA level in tumor tissues; (e) apoptosis and vessels in tumor tissues detected by TUNEL assay (upper) and CD31 staining (lower).

A C26 xenograft animal model was also utilized to test the antitumor efficacy of CLPP/VSVMP mRNA complex *in vivo*. The tumor growth curves and images of C26 xenograft tumors of each group are presented in Fig. 6a and b. According to our results, intratumorally injection of CLPP/VSVMP mRNA resulted in a significant inhibition of xenograft tumor growth compared with control groups. The weight of the tumors in each group is presented in Fig. 6c. Comparing with NS treatment group (0.7 ± 0.1 g) and CLPP group (0.6 ± 0.1 g), mRNA complex caused a statistically significant reduction in tumor weight (0.2 ± 0.1 g, *P* < 0.01). Meanwhile, it could be observed that CLPP/VSVMP mRNA complex showed comparable anti-cancer ability with liposome delivered VSVMP plasmid group (0.2 ± 0.1 g). These results suggest that intratumorally injection of CLPP/VSVMP mRNA complex could efficiently inhibit the growth of subcutaneous xenograft of C26 colon cancer model. Its anti-cancer capacity *in vivo* was equivalent to conventionally used plasmid formulation, which was consistent with the *in vitro* data.

The expression of VSVMP in tumor tissues from both models were confirmed by qPCR analysis. According to our results (Fig. 5e and 6d), significant enhanced VSVMP mRNA levels were detected in CLPP/VSVMP mRNA complex treatment groups, with over 20 folds in metastases mode and nearly 4 folds in xenograft mode comparing to control group, respectively. The high mRNA levels being detected indicated that VSVMP mRNA was efficiently delivered into tumor cells by liposome–protamine complex. However, we also observed that in xenograft model, much higher mRNA level was detected in CLP delivered VSVMP plasmid group (400 folds, Fig. 6d), suggesting a more sustained expression behavior than mRNA delivery form.

**Fig. 7:**
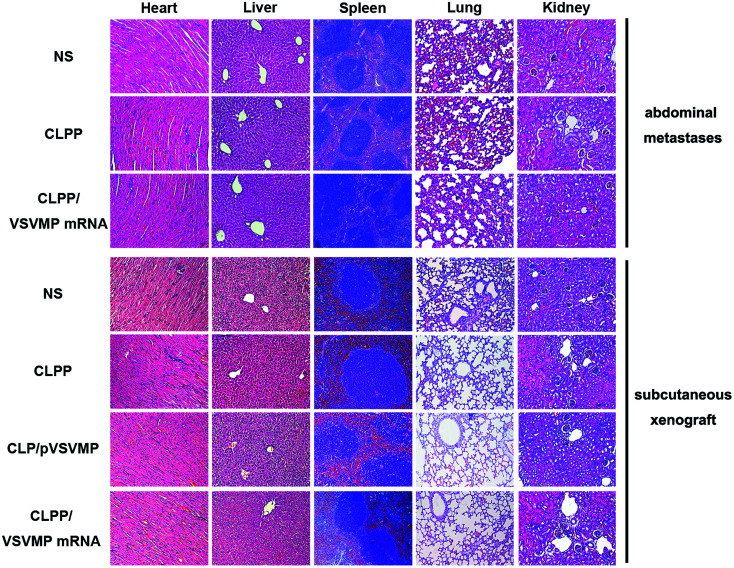
HE analysis of main organs from each treatment group in both models. No significantly pathological changes were observed in heart, liver, spleen, lung, or kidney.

*In vivo* anti-tumor mechanisms of CLPP/VSVMP mRNA complex in above two models were further studied by TUNEL assay and CD31 staining. As shown in Fig. 5f and 6e, treatment with mRNA complex induced a significantly increase in apoptosis within tumor tissues compared to other groups as determined by the TUNEL assay. These performances could be spotted in both animal models, suggesting that VSVMP mRNA was efficiently delivered by liposome–protamine complex and expressed *in vivo*. In addition, the CLPP/VSVMP mRNA complex treatment groups from both models also showed anti-angiogenesis effects in tumors compared to other groups as determined by CD31 staining (Fig. 5f and 6e). The micro-vessel density characterized by CD31 positive staining was significantly attenuated in the mRNA complex treatment group, when compared with NS, liposome–protamine alone, or VSVMP plasmid group. Our results suggested that CLPP/VSVMP mRNA complex could also inhibit tumor growth through anti-angiogenesis mechanism. Furthermore, the *in vivo* side effects of CLPP/VSVMP mRNA complex on other organs were examined through HE analysis. As shown in Fig. 7, no significantly pathological changes in heart, liver, spleen, lung, or kidney were observed. Overall, our data suggested that CLPP/VSVMP mRNA complex are capable of treating C26 colon cancer by inducing apoptosis and angiogenesis inhibition without high safety.

## Discussion

As an alternative form of therapeutic gene, IVT mRNA have been applied in several gene therapy studies and biomedical researches. In previous studies, we have evaluated the anti-cancer ability of non-viral vector delivered suicide gene VSVMP in a form of plasmid DNA. In this work, a IVT mRNA form of VSVMP gene was delivered by liposome–protamine complex, and its anti-cancer potential was evaluated both *in vitro* and *in vivo*. Our results showed that liposome–protamine complex could efficiently delivery VSVMP mRNA into C26 colon cancer cells with high efficiency. The mRNA–liposome–protamine complex could strongly inhibit the growth of tumor cell both *in vitro* and *in vivo* though inducing apoptosis. Our results demonstrated that liposome–protamine complex delivered VSVMP mRNA was as potent as its plasmid counterpart, showing strong potential in further colon cancer therapy.

In this work, a liposome–protamine formulation was utilized for the delivery of IVT mRNA. Within this formulation, cationic liposome act as a vector for gene transfection while protamine was taken advantaged to condense naked mRNA. It has been widely reported that nucleic acid condensed by protamine condensed was protected from nuclease degradation inside the lysosomes/endosomes, resulting in high expression efficiency.^31^ In previous reports, IVT mRNA of different genes were mainly delivered either by protamine alone^20,39^ or cationic vectors.^40,41^ As mentioned above, both strategies seem to be applicable in mRNA delivery.^11,34,39^ However, few reports have involved combining these two components into one formulation for mRNA delivery. In contrast, we made an attempt by using liposome–protamine complex to take advantage of the two. In our study, the IVT mRNA was condensed with protamine followed by liposomal delivery. Using EGFP coding mRNA as a reporter gene, the delivery efficiency reached 47% in 24 hours post-transfection. Meanwhile, a significant VSVMP mRNA level was detected comparing to untreated group, suggesting a high delivery efficiency. Our results also showed that barely no EGFP expression could be detected from protamine–mRNA complex without cationic liposome. Meanwhile, although cationic liposome alone could also deliver EGFP mRNA in a lower level, the fluorescent intensity was much weaker than liposome–protamine complex. The results suggested that in our design, cationic liposome is critical for mRNA delivery while condensed mRNA by protamine is not capable of accessing into cytoplasm alone. Comparing to protamine, cationic liposomes might provide better protection for cargoes from degradation in either culture medium or serum environment. On the other hand, our result also indicated that protamine is necessary for delivering mRNA with high efficiency. The condensing and complexing abilities of protamine have long been recognized.^42,43^ These properties are not limited to mRNA but also applicable for other nucleic acids. According to our previous results (data not shown), enhanced plasmid transfection was observed when protamine was added, suggesting an optimizing strategy for plasmid DNA-based gene therapy. Thus, these results indicated the liposome–protamine complex formulation to be a practicable and an alternative strategy for mRNA delivery. Our work demonstrated that potentially higher delivery capacity could be achieved by combining these two. Despite of these, the length of mRNA should be taken into consideration when delivered with protamine–liposome complex. In this study, a EGFP encoding mRNA with a total length of 996 bases was used as reporter gene. Thus, there is high possibility that VSVMP mRNA with a short length (690 nucleotides) was delivered under similar efficiency. However, delivering capacity might be limited when therapeutic gene is longer. Thus, proper optimization of liposome–protamine combination might be necessary when different therapeutic genes are employed.

In previous works, the anti-cancer capacities of VSVMP encoding plasmid has been studied in several tumor models.^10,37^ The cell apoptosis inducing and anti-proliferation capacity of VSVMP gene have been well characterized, demonstrating strong potential in cancer gene therapy. In our study, the anti-cancer properties of VSVMP gene in plasmid and mRNA form were compared. According to our results, after receiving the same amount of nucleic acids, significantly higher VSVMP mRNA level was detected in mRNA group *in vitro*. Meanwhile, under the same condition, mRNA group was superior to DNA group in both apoptosis inducing and anti-proliferation study, these enhanced anti-cancer effect resulted in strongest inhibition on the cell viability of C26 cells (Fig. 4b). Therefore, based on results above, our study has demonstrated obvious advantage in mRNA delivery form. This might be explained by several reasons. First, plasmid form carries much more nucleic acid elements than mRNA including promoter regions, protein tags and resistance tags. In contrast, fewer elements result in fewer burdens and higher delivery efficiency. For the transfection of C26 cells by cationic liposome, our previous work has indicated a transfection efficiency of approximate 10% with plasmid DNA,^44^while that for mRNA form (even without protamine) is increased (15%). Meanwhile, by delivering the same EGFP gene, liposome–protamine–mRNA complex resulted in a efficiency of more than 40% in various cell types. A second reason might be taken into consideration that it takes more steps for plasmid DNA in translating process than mRNA and nuclear localization of mRNA is not required before starting protein expression. Translocation of exogenous DNA through the nuclear membrane is a major concern of gene delivery and expression.^45^ Conventional delivery methods usually suffer from the inefficient nuclear uptake of plasmid DNA introduced into the cell.^46^ However, therapeutic gene in mRNA form has got across this stage naturally. Moreover, different vectors used for DNA and mRNA delivery might also result in diverse intracellular degradation pathways.^18^ For these reasons, mRNA complex might act faster than pDNA counterparts under certain circumstances and demonstrating better biomedical effects. However, in another aspect, factors such as production difficulty, costs as well as the function of elements should not be omitted when making selection between these two since plasmid DNA might be more convenient for scaled production.

Despite these *in vitro* results, the efficacy difference between pDNA complex and mRNA complex in our *in vivo* experiment on C26 xenograft model was not significate enough as predicted. Furthermore, at the endpoint of treatment, much higher level of VSVMP mRNA was detected in pDNA group. As mentioned above, it can be inferred that the expression of plasmid DNA is slower than mRNA, and their degradation behaviors might be varied, which might result in more sustained expression behavior. Meanwhile, repetitiously administration might strengthen this effect. Nevertheless, mRNA gene delivery form might effective avoid the “backbone effect” of plasmid DNA, which refers to the cytotoxicity caused by empty plasmid itself. In previous gene therapy reports including VSVMP, the effect of empty plasmid/gene vector could be observed occasionally.^8,10,44^ One possible explanation for this is that cationic agent/bacterial DNA complexes may elicit adaptive immune response under certain circumstances.^47^ This phenomenon has also been observed on viral vectors.^48^ Thus, comparing to mRNA complex, although similar anti-cancer capacities were achieved in DNA group *in vivo*, it is questionable that to what extend it was a consequence of VSVMP coding sequence but plasmid backbone. What's more important, it makes us further worry about the side effects caused by “backbone effect” of plasmid DNA apart from therapeutic outcome, while this by-product could not be clarified easily. For example, in previous reports regarding colon cancer gene therapy, various therapeutic genes such as VSVMP,^10,37^ survivin-T34A^8,9^ and IL-12 (ref. 44 and 49) have been applied in plasmid form. However, no matter whether the therapeutic effects were results of cell apoptosis inducing or microenvironment immune response stimulating, the researchers still need to answer those questions above so as to scientifically assess the mechanisms. Therefore, mRNA delivery form provides an alternative solution for it and potential debate, and our present study suggested an optimized strategy for VSVMP-based gene therapy research. Anyway, despite the *in vitro* results, further optimization of mRNA formulation and administration strategy are still necessary in our future study to better reveal the potential superiority of mRNA over plasmid DNA *in vivo*.

## Conclusions

In this work, an *in vitro* transcription mRNA encoding VSVMP gene was successfully delivered by cationic liposome–protamine complex. The liposome–protamine complex delivered VSVMP mRNA could efficiently inhibit the growth of C26 colon carcinoma both *in vitro* and *in vivo* with high safety. Our results demonstrated the potential capacity of liposome–protamine complex in non-viral gene delivery and offered an alternative strategy for colon cancer gene therapy.

## Conflicts of interest

There are no conflicts to declare.

## Supplementary Material
